# Hematological and Cytogenetic Effects of X-rays in Cardiac Unit Workers and Catheterization Patients

**DOI:** 10.7759/cureus.53593

**Published:** 2024-02-05

**Authors:** Haliz Hussein, Asaad Alasady, Khairi MS. Abdullah

**Affiliations:** 1 Department of Anatomy, Histology, and Biology, College of Medicine, University of Duhok, Duhok, IRQ; 2 Department of Water Resources, College of Engineering, University of Duhok, Duhok, IRQ

**Keywords:** chromosomal aberrations, damage cells, x-ray, hematological parameters, cardiac catheterization

## Abstract

Introduction

X-rays are widely used in medicine for diagnosis and treatment. Such beneficial uses may cause potentially hazardous situations for patients and workers in the cardiac catheterization laboratory. The present study aims to estimate the radiation dose scattered in different parts of the catheterization units and doses absorbed by workers in this unit, and patients who underwent cardiac catheterization procedures to evaluate all changes in hematological parameters and damaged cells (the cells that contain a number of chromosomal aberrations) after exposure to radiation at Azadi Teaching Hospital in the Duhok City of Iraq.

Methodology

The study was conducted in one year and involved 19 male workers chronically exposed to X-ray machines in the cardiac catheterization laboratory, and 45 patients, 20 males and 25 females, who have been exposed to lower doses of X-ray during the cardiac catheterization process. There were 32 healthy individuals, 19 males and 13 females, as a control. Scattered radiation was calculated using an area monitoring detector. Optically Stimulated Luminescence (OSL) dosimeter and Flat Panel Detector (FPD) were used to calculate absorbed doses by workers and patients, respectively. Twelve hematological parameters before and after radiation were examined between study groups; the cytogenetic effects, damaged cells, and chromosomal aberrations of the white blood cells of workers, patients in the catheterization unit, and individuals of the control group were analyzed.

Results

The results showed that the scattered X-rays in the catheterization unit after one year of continuous detection did not change significantly compared to the data before the start of the trial. The results of all blood parameters looked to be significantly different (p<0.05) compared to the controls but within the normal range. There is no significant difference (p>0.05) in corpuscular hemoglobin, white blood cells, red distribution width, and neutrophil values for workers after one year of exposure as compared with the control. Also, there was no significant difference (p>0.05) in white blood cells, neutrophils, and monocyte values for patients after the operation. The current study showed the damaged cells in workers were significantly different compared to the control. At the same time, the differences were non-significant for all workers (p=0.0962) after one year of exposure. The differences in damaged cells in patients were highly significant after the operation (p=0.0003). The present study demonstrated that the inductions of dicentrics, acentric, chromosome break, and ring chromosomes in human lymphocytes were intimately related to the irradiation dose.

Conclusions

The present study found that the scattered X-rays in the catheterization unit after the end of the experiment did not change significantly. The current study also revealed that the exposure to X-rays had no significant effects on the blood indicators of workers and patients in the catheterization unit, whereas the damaged cells in patients did not change significantly compared with the control group at the beginning of the experiment. In patients, these cells were increased after the operation but were present at a high level in the workers, as compared with controls. The damaged cells in workers remained constant from the beginning of the experiment till the end. Finally, patients had increased damaged cells after the end of the trial period compared to workers.

## Introduction

X-rays are a form of ionizing radiation that has been found useful in medical imaging; at the same time, excessive exposure to radiation may damage living tissues and organs, depending on the radiation dose [1]. Ionizing radiation can cause various forms of cell damage, including the possibility of an increased incidence of chromosomal aberrations. The cytotoxic effects of low-dose ionizing radiation in occupationally exposed radiation workers have been recorded in several previous studies [2,3].

Cardiac catheterization is a procedure used to check the heart for blocked arteries and valve abnormalities, in which a cardiologist inserts a long, thin catheter into an artery in the arm, neck, or groin, threads it into the heart, and then injects contrast material. The arteries are dyed, and fluoroscopy is done as the contrast material moves through the heart's, chambers, valves, and major vessels [4]. 

Fluoroscopy is an ideal continuous X-ray machine used in cardiac catheterization laboratories for medical imaging. It is often used in many different investigations and procedures in the diagnostic or treatment proceeding of heart disease patients [5]. In front of fluoroscopy advantages, it involves some risks and may cause potentially hazardous situations for the patients and the workers, as do other X-ray operations. The radiation dose and the time of operation are the most important controlled parameters that the patient is subjected to, which vary from one patient to another depending on the individual procedure [6].

Analyses of hematological parameters, such as blood cell counts are often used in routine medical examinations. Hematopoietic cells are highly sensitive to ionizing radiation. Long-term exposure to low doses of ionizing radiation may adversely affect human cells, especially peripheral blood cell counts [7]. A study conducted in the Duhok City, Kurdistan region of Iraq [8] showed that the low level of X-ray irradiation, during the catheterization process, significantly decreases the erythrocyte (RBC) counts, plasma hemoglobin (Hb) and WBC counts in blood post-irradiated, and there are no significant changes observed between pre- and post-irradiated levels in red blood cell distribution width (RDW) and mean corpuscular volume (MCV) sizes. On the other hand, a cross-sectional study [9] in which subjects with more than two years of experience working in a catheterization laboratory (cath lab), who have been exposed to continuous radiation demonstrated that changes in blood function due to ionizing radiation are affected by the duration of exposure to radiation and the effective dose.

In addition, a study conducted in southern Nigeria [10] found lower counts of white blood cells (WBC), neutrophils, and lymphocytes, and a higher range of abnormal forms of blood cells in workers exposed to continuous radiation with more than two years of experience in the cath lab. compared to controls. Furthermore, an Indonesian study [11] reported higher red blood cell and monocyte counts in radiation workers at several governmental hospitals in Indonesia compared to the control group. A study [9] in a Palestinian population noted perturbation of low and high mean values of hematocrit and hemoglobin in some medical photographers in the X-ray unit. Changes in the hematological parameters of radiation workers can be used to predict radiation damage. Moreover, absorbed energy can form reactive oxygen species, chemical bond breaking, and ionization of various biologically essential macromolecules such as DNA, lipid concentration, and proteins. The DNA damage induced by irradiation involves strand breaking or chromosomal abnormalities that can lead to mutation [12]. There are no studies in Iraq and neighboring regions. Therefore, the present study aimed to estimate the radiation dose scattered in different parts of the catheterization units and to evaluate and analyze the effect of low-dose X-rays during the catheterization process on the hematological parameters, damaged cells, and chromosome aberrations after exposure to radiation for medical radiographers and patients who undergo the catheterization process.

## Materials and methods

Study population

This cohort study was conducted in the Cardiac Catheterization Center of Azadi Teaching Hospital in Duhok City, Iraq after obtaining approval from the ethics committee. The study included 19 male medical radiation workers occupationally exposed to low doses of ionizing radiation in the unit of cardiac catheterization with age ranges 27-47 years, with a mean of 37.5±4.12. Also, it included 45 patients, 25 males and 20 females, who underwent the cardiac catheterization process with ages ranging from 30 to 58 years, with a mean of 44 ±1.97. At the same time, 32 control groups, 19 male and 13 female members, with an age range of 29-54 years, with a mean of 41.5 ± 1.76, were chosen from non-radiation workers. All participants in this study were given their informed consent. After being informed of the study scope and experimental details we recorded all the information required for all participants.

Determination of X-ray doses

To determine the scattered X-ray doses in different parts of the catheterization laboratory an Area Monitoring detector was used, and the OSL InLight dosimeter (LANDAUER Scitek, Glenwood, USA) was used for recording the X-ray absorbed doses by workers, whereas for determining the X-radiation absorbed doses, by patients who underwent cardiac catheterization at the cardiac catheterization lab of Azadi Teaching Hospital in Duhok city, a Flame Photometric Detector (FPD) (SIEMENS AG) was used. Consequently, all detectors were placed in designated places in the catheterization center.

Collection of blood samples

Blood samples were taken from all participants in the present study according to standard protocols. From December 2021 to December 2022, Venous blood was taken with disposable syringes from each individual 5 ml of blood was drawn before and after exposure to X-rays, for workers and patients, whereas for the control group, the blood was drawn from each individual before and after the experiment.

Hematological study

Blood sample 2-3 ml was transformed into ethylenediamine tetraacetic acid (EDTA) tubes for the laboratory examinations that covered a Complete Blood Count (CBC) to account for a number of all components in the blood. The CBCs for all workers, patients, and control groups were measured by an automatic hematology analyzer (Swelab Alfa basic, Boule medical) (SN: 11018) at the laboratory of Azadi Teaching Hospital at the start of the experiment. To investigate the effect of radiation on the CBCs of workers, blood samples were taken from 19 male medical radiation workers aged 30-54 years who were exposed to low radiation (X-rays) 0.01-23.45 mSv with a mean 5.034±0.860 mSv for one year. At the same time, blood samples were drawn from healthy people aged 29-54 years in 32 individuals, including 19 males and 13 females after one year as control. Also, blood samples from 45 patients who underwent the cardiac catheterization process, 20 males and 25 females aged between 30-58 years, were collected before the operation and after 48-72 hrs of the operation to study the effect of absorbed radiation on the blood.

Cytogenetic study

To investigate the effect of radiation on the genetic materials (chromosomes) of workers, blood samples of 1-2 ml from 19 male workers in the cardiac catheterization lab when starting the experiment, and after one year all samples were transformed into a heparinized tube for cytogenetic study. As well as blood samples from 45 patients who underwent the cardiac catheterization process in the cardiac catheterization center were collected before the operation and after 48-72 hrs of the operation. Furthermore, from 32 individuals healthy people aged 25-58 years, including 19 males and 13 females, blood samples were drawn at the beginning of the experiment and after one year as control samples by disposable syringes and transformed into the heparinized tube to study the effect of X-ray on chromosomal aberrations. Each heparinized tube was transported immediately to the laboratory for culturing with RPMI-1640 medium (Thermo Fisher Scientific, USA). The study subject was at 37 °C for 72 hours and chromosomes were harvested and stained by G-banded, and the abnormalities were detected according to International System for Human Cytogenetic Nomenclature (ISNC) [13]. Labeling was done with a unique number for each subject for cytogenetic analysis at the Scientific Research Center College of Medicine University of Duhok. The karyogram was obtained by computer-assisted analysis and cytovision program [14].

Statistical analysis

The hematological measurements of study groups were presented in mean ± SD. The comparisons of hematological parameters between before and after radiation in each group were examined in a paired t-test. The significant level of difference was determined in a p<0.05.

The comparisons of damaged cells in the control, patient, and staff were done in an independent t-test. The damaged cells before radiation exposure and after radiation exposure were analyzed by paired t-tests. The significant level of difference was determined in a p <0.05. The statistical calculations were performed by JMP Pro 14.3.0 (https://www.jmp.com/en_us/home.html).

## Results

Direct and scattered radiation doses

The results showed that the scattered X-ray absorbed by workers at the beginning of the study was 0.01-14.21 mSv with a mean of 2.90 ± 0.48 mSv, whereas after one year ranged from (0.01 to 23.45) mSv with a mean of 5.034±0.860 mSv was significantly changed at (p<0.020). However, depending on the measured direct radiation dose on the patient by an FPD during the operation ranging from 124 to 2588 mSv with a mean of 347.77 ±120.63 mSv for the average operation time of 4.78± 0.37 min.

Hematological effects of X-ray

The statistical analysis for comparisons between the hematological parameters of the control group n=19 males aged 30-48 years before and after one year of the experiment are illustrated in Table 1. The results showed a significant difference (p=0.0395) in Hb which increased but thankfully within the normal range. While the RBC and Neutrophils decreased at (p=0.0062, 0.0139), respectively also within the normal range. While slight changes in other blood parameters were not significant.

**Table 1:** Mean ± SD for hematological parameters before and after one year of the trial between the control and worker groups *Change within the normal range; n, number; SD, standard deviation; RBC, red blood cell; PCV, positive crankcase ventilation; MCV, Mean Corpuscular Volume; MCH, mean corpuscular hemoglobin; MCHC, mean corpuscular hemoglobin concentration; RDW, Red cell distribution width; WBC, white blood cells.

			Study groups		
		Before X-ray exposure		After X-ray exposure	
Hematological parameters		control	Workers	control	Workers
	Ref. Range	n=19	n=19	n=19	n=19
Hemoglobin g/dl	13.0 – 17.0	12.37 ± 0.58	15.28±1.00*	12.75 ±0.89 *	15.16 ±0.96 *
RBC x10¹² /l	4.5 – 6.1	5.27 ± 0.32	4.93 ±0.66 *	4.73 ±0.68*	5.30 ±0.49 *
PCV %	36.1- 50.3	37.71 ± 1.81	41.92 ±2.65*	38.14 ±1.78 *	44.32 ±3.41 *
MCV fl	80.0 – 100.0	80.18 ±3.47	81.68 ±11.67*	83.81 ±12.59 *	85.36 ±3.76 *
MCH g/dl	27.0 – 33.0	28.38 ±2.13	28.63 ±2.68*	28.36 ±2.15 *	28.85 ±2.77*
MCHC g/dl	33.0 – 37.0	36.45 ±2.48	36.34 ± 0.86 *	36.44 ±2.49 *	34.11 ±1.31 *
RDW %	11.6 – 14.6	12.43 ±0.75	11.52 ±0.44*	12.31 ±0.83 *	12.22 ±0.47*
Platelet countx10ᶺ9/l-	150 – 450	268.07±43.06	207.58 ±53.15*	261.79 ±49.75*	229.95 ±71.62 *
WBC x10ᶺ9/l	4.0 -11.0	7.05 ±2.00	6.54 ±1.56 *	7.02 ±1.94 *	7.06 ±2.10 *
Neutrophils 10ᶺ9\µl	2.0 – 7.0	4.78 ±1.66	3.54 ±0.68*	4.56 ±1.63 *	4.30 ±1.60 *
Lymphocytes 10ᶺ9/µl	1.0 – 3.5	3.09 ±1.19	3.51 ± 0.81 *	3.05 ±1.18*	3.60 ±1.07
Monocytes 10ᶺ9/µl	0.2 – 1.0	0.71 ±0.19	0.49 ±0.12 *	0.64 ±0.21 *	0.51 ± 0.15 *

In contrast, the comparisons between the hematological parameters of the cardiac unit workers before the starting of the trial of exposure to X-ray and controls were illustrated in Table 1. The results showed a significant difference in Hb, positive crankcase ventilation (PCV), and MCV, which increased at p<0.0001 and p=0.0100, respectively but within the normal range. While for RDW%, platelet count, neutrophil, monocyte, and RBC the changes were significantly declined at p<0.0001, and p=0.0005, 0.0077, 0.002, and 0.0258, respectively, also within the normal range except for mean corpuscular hemoglobin (MCH), mean corpuscular hemoglobin concentration (MCHC), WBC, and lymphocyte were non-significant change (p>0.05).

The hematological parameters for workers after one year of exposure to X-rays that compare with the control group are shown in Table 1. The results revealed a significant difference between workers and control groups in all, Hb, PCV, RBC, and MCV which increased at (p<0.0001, 0.0084, 0.0016), respectively but within the normal range, the results of the present study show a significant decreased in MCHC, and monocytes, at (p=0.0009 and 0.0290), respectively but within the normal range. While for RDW%, platelets, and neutrophils, the difference non-significantly decreased (p>0.05) and non-significantly increased in MCH, and WBC, respectively. In addition, for lymphocytes the difference was not significantly increased than the normal range (p=0.1887).

Furthermore, the comparisons of the hematologic parameters of workers in catheterization units before and after one year of exposure to X-rays are shown in Table 1. The results showed a significant difference in PCV, RBC, RDW%, MCV, and neutrophils, which increased at (p=0.0046, <0.0001, and 0.0322), respectively but within the normal range, whereas lymphocytes were non significantly increased than the normal range at (p=0.9288). While MCHC significantly declined at (p< 0.0001) but within the normal range. On the other hand, Hb was non-significantly decreased at (p>0.05), however, for MCH, platelets, WBC, and monocytes were non-significantly increased at (p>0.05).

The statistical analysis for comparisons of hematological parameters between the patients before exposure to radiation (before operation) and the control group is shown in Table 2. The changes in hematological parameters significantly increased in Hb, PCV, and MCV at (p=0.0332, 0.0420, and <0.0001), respectively but within the normal range. Moreover, for WBC and neutrophils, the changes were not significantly increased at p=0.1493 and 0.7553, respectively; also for MCH the changes were not significantly declined at (p=0.8518). Whereas, for RDW%, platelet count, MCHC, lymphocyte, RBC, and monocytes, the change significantly declined at (p=0.0004, 0.0328, and <0.0001), respectively but within the normal range.

The comparisons of hematological parameters of patients before operation, and after the operation are shown in Table 2. The results showed a significant decrease in Hb, RBC, PCV, MCV, and WBC count but this decreased within the normal range at (p<0.0001), and at (p=0.0008) for RDW%, While it decreased non-significantly for monocytes at (p=0.1341) and increased at (p=0.073) for neutrophils, but the changes were within the normal range may be due to short time of exposure that ranged from 1.1-18.3 minutes with mean 5.13±3.78. Furthermore, for MCH, MCHC, and lymphocytes the change was significantly increased at (p<0.0001) and at (p=0.0043) for platelets but within the normal range.

**Table 2:** Mean ± SD for hematological parameters of patients before the operation compared with the control group and patients after radiation An independent t-test and paired t-test were performed for statistical analyses. *Change within the normal range; n, number; SD, standard deviation; T, Total; M, Male; F, Female; n, number; SD, standard deviation; RBC, red blood cell; PCV, positive crankcase ventilation; MCV, Mean Corpuscular Volume; MCH, mean corpuscular hemoglobin; MCHC, mean corpuscular hemoglobin concentration; RDW, Red cell distribution width; WBC, white blood cells.

		Study groups		
Hematological parameters	Ref. Range	Control	Patients before X-ray exposure	Patients After X-ray exposure
		n=32	n=45	n=45
Hemoglobin g/dl	M.13.0 -17.0	T.12.39±0.75	T. 13.05 ±1.27 *	T. 12.16±1.19*
	F. 12.0 -15.5	M. 12.37 ±0.69	M. 13.7±1.12	M.12.85±1.29
		F.12.42±0.81	F.12.53 ±0.114	F. 11.48±1.09
RBC x10¹² /l (before)	M. 4.5 - 6.1	T. 5.11±0.23	T. 4.90 ±0.55*	T. 4.22±0.54*
	F. 4.2-5.4	M. 5.27 ±0.32	M.5.14±0.48	M. 4.56±0.54
		F.4.95±0.14	F. 4.70 ±0.52	F.3.88±0.55
PCV% (before)	M. 36.1- 50.3	T. 37.22±1.85	T. 39.97 ±4.19*	T.35.46±4.39 *
	F. 36.1-44.3	M. 37.71 ±1.81	M. 41.64±4.26	M.37.61±5.04
		F.36.73±1.89	F. 38.31±3.55	F.33.31±3.75
MCV ( fl)	M. 80.0 -100.0	T. 82.44 ±5.70	T. 83.89 ±6.07 *	T. 80.07±6.48 *
	F. 80.0 -100.0	M. 80.18 ±3.47	M. 83.16±7.65	M.79.96±8.40
		F.84.7±7.94	F. 84.47±4.51	F. 80.18±4.56
MCH (pg)	M. 27.0 -33.0	T. 31.08±7.53	T. 27.60 ±2.13 *	T.30.53±4.04*
	F. 27.0 -33.0	M. 28.38 ±2.13	M. 27.48±2.50	M.29.22±2.90
		F. 33.79 ±12.94	F. 27.40 ±2.33	F. 31.84±5.19
MCHC g/dl	M. 33.0 - 37.0	T. 35.85±2.95	T. 32.85 ±0.93 *	T.35.68±3.16 *
	F. 33.0 - 37.0	M. 36.45 ±2.48	M.32.90±1.57	M.35.18±2.86
		F. 35.26±3.43	F.32.81±0.12	F.36.18±3.46
RDW%	M. 11.6 -14.6	T. 12.47±0.82	T. 12.37 ±0.45 *	T.11.71±1.34 *
	F. 12.2-16.1	M. 12.43 ±0.75	M. 13.12±0.25	M.12.17±1.75
		F. 12.51 ±0.90	F. 11.63±0.66	F.11.25±0.94
Platelet count x10ᶺ9/l	M. 150 - 450	T.255.77±55.39	T. 232.11±68.19 *	T. 232.52±61.3*
	F. 150 - 450	M. 268.07 ±43.06	M. 236. 35±77.55	M.240.95±70.13
		F. 243.47±67.73	F. 227.72±58.83	F.224.09±52.47
WBCx10ᶺ9/l	M. 4.0 -11.0	T. 7.36±2.26	T. 7.90 ±1.76 *	T. 7.14±1.71*
	F. 4.5-11.0	M. 7.05 ±2.00	M. 8.66±2.05	M.7.9±1.86
		F. 7.67±2.53	F. 7.14 ±1.47	F.6.38±1.57
Neutrophils 10ᶺ9\µl	M. 2.0 -7.0	T. 4.44±1.88	T. 4.66 ±1.35*	T.4.97±1.41 *
	F. 2.0 -7.0	M. 4.78 ±1.66	M.4.76±1.60	M.5.09±1.59
		F. 4.11±2.11	F. 4.57±1.11	F.4.86±1.23
Lymphocytes 10ᶺ9 /µl	M. 1.0 - 3.5	T. 3.6 ±0.9	T. 2.46 ±0.61 *	T. 2.80±0.9*
	F. 1.0 - 3.5	M. 4.09 ±1.19	M. 2.62±0.68	M. 2.94±0.99
		F. 3.11 ±0. 61	F.2.31±0.54	F.2.67±0.81
Monocytes 10ᶺ9/µl	M. 0.2 -1.0	T.0.68±0.23	T. 0.36 ±0.14 *	T. 0.33±0.17*
	F. 0.2 -1.0	M. 0.71 ±0.18	M. 0.42±0.16	M. 0.38±0.20
		F. 0.66 ±0.29	F. 0.31±0.12	F.0.29±0.14

Furthermore, Table 3 shows the comparisons between hematological parameters for patients (n=20) and workers (n=19) before exposure to radiation in male cases. The results revealed a significant difference in Hb, MCHC, MCH, lymphocytes, and monocytes, which significantly increased at p<0.0001, 0.0090, 0.0002, and 0.0478, respectively but within the normal range. Also, for PCV the change was non-significantly increased at (p>0.05). While the changes in RDW%, RBC, WBC count, and neutrophils were significantly decreased at (p<0.0001, 0.0015, and 0.0050), respectively, but within the normal range, and non-significantly decreased at (p>0.05) for MCV and platelet. 

**Table 3:** Means ± SD for the hematological parameters of patients and workers while starting the experiment, and After radiation exposure An independent t-test was performed for statistical analyses. *Change within the normal ranges. SD, Standard deviation; n, number; SD, standard deviation; RBC, red blood cell; PCV, positive crankcase ventilation; MCV, Mean Corpuscular Volume; MCH, mean corpuscular hemoglobin; MCHC, mean corpuscular hemoglobin concentration; RDW, Red cell distribution width; WBC, white blood cells.

Study groups					
Hematological parameters	Ref. Range	Before X-ray exposure		After X-ray exposure	
		Patients n=20	Workers n=19	Patients n=20	Workers n=19
Hemoglobin g/dl	13.0 – 17.0	13.7 ±1.12	15.28 ±1.00*	12.85±1.30	15.16 ±0.96*
RBC x10¹² /l (before)	4.5 – 6.1	5.14 ±0.48	4.93 ±0.66 *	4.56±0.54	5.30 ±0.49*
PCV% (before)	36.1- 50.3	41.64 ±4.26	41.92 (2.65) *	37.61 ±5.04	44.32 ±3.41*
MCV ( fl)	80.0 – 100.0	83.16 ±7.66	81.68±11.67 *	79.96 ±8.41	85.36 ±3.76*
MCH (pg)	27.0 – 33.0	27.48 ±2.51	28.63 ±2.68 *	29.22 ±2.90	28.85 ±2.77 *
MCHC g/dl	33.0 – 37.0	32.90 ±1.57	36.34 ±0.86 *	35.18 ±2.87	34.11 ±1.31*
RDW% (before)	11.6 – 14.6	13.12 ±0.25	11.52±0.44*	12.17 ±1.75	12.22±0.47*
Platelet count x10ᶺ9/l	150 - 450	236.35 ±77.55	207.58±53.15 *	240.95±70.14	229.95 ±71.62*
WBCx10ᶺ9/l (before)	4.0 -11.0	8.66 ±2.05	6.54±1.56*	7.9 ±1.86	7.06 ±2.10 *
Neutrophils(before)10ᶺ9\µl	2.0 – 7.0	4.76 ±1.61	3.54 ±0.68*	5.09 ±1.59	4.30 ±1.60 *
Lymphocytes 10ᶺ9 /µl	1.0 – 3.5	2.62±0.68	3.51 ±0.81*	2.94 ±0.99	3.60 ±1.07*
Monocytes (before)10ᶺ9/µl	0.2 – 1.0	0.42±0.16	0.49±0.12 *	0.38 ±0.20	0.51±0.15*

The comparisons between hematological parameters for patients and workers after exposure to radiation in the male case are illustrated in Table 3. The results showed a significant difference in Hb, RBC, PCV, MCV, lymphocytes, and monocytes which significantly increased at p<0.0001, 0.0233, 0.0549, 0.0006, respectively but within the normal range, and also, the change was non significantly increased at p>0.05 in RDW%, and non-significantly decreased in MCH, MCHC, platelets, WBC, and neutrophils.

Cytogenetic effect of X-ray

Cytogenetic analysis was performed on peripheral blood lymphocytes of all male workers (n=19) with different ages ranging from 27 to 47 years with a mean of 37.5 ±4.12, whereas, the age of the control group (n = 19), the age ranges from 30-54 years with mean 40.11 ± 6.13.

Statistical analysis for comparisons of damaged cells and the percentage of chromosomal aberrations between workers and the control group at the beginning of the trial is shown in Table 4. Statistical analysis shows significant differences (p=0.0083) in damaged cells between the control and workers groups, with a mean of 1.47 ± 0.70 and 2.42 ± 1.30, respectively.

**Table 4:** Comparisons of damaged cells and percentage of chromosomal aberrations between control and workers at the start of the experiment and after one year. Any cell containing one or more chromosome aberrations is counted as one damaged cell.

Chromosomal aberrations	Study groups			
	Before exposure to X-ray		After exposure to X-ray	
	Control (n=19) % damage cells	Workers (n=19) %damage cells	Control (n=19) % damage cells	Worker(n=19) % damage cells
Dicentric Chromosome	3	6	3	24
Acentric Chromosome	15	16	16	25
Chromatid Gap	2	1	2	1
Chromosome break	0	15	0	15
Chromosome interchange	0	0	0	0
Ring Chromosome	10	8	13	10
Damage cells (%) mean (SD)	1.47 (0.70)	2.42 (1.30)	1.79 (0.61)	2.95 (0.91)
P-value	0.0083		<0.0001	

**Figure 1:**
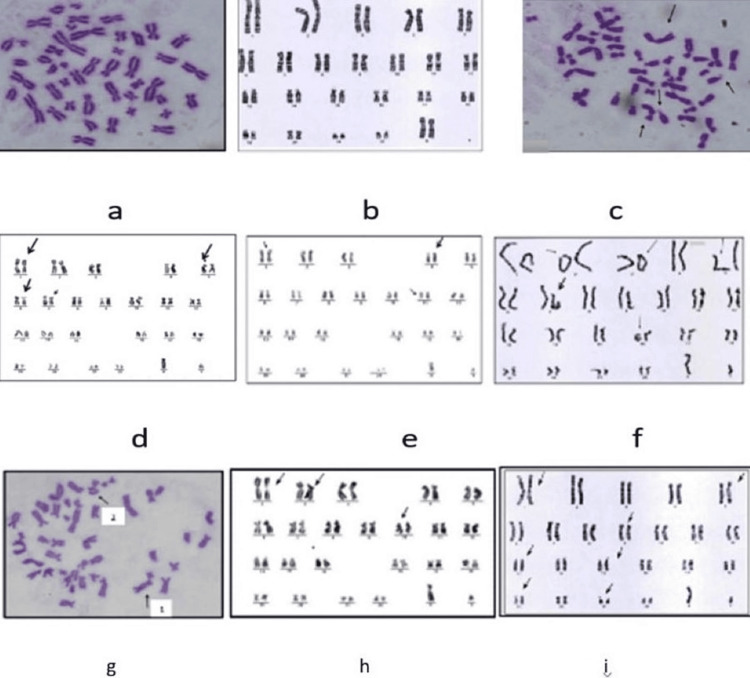
Karyotype of two individuals in the control groups and metaphase of structural abnormality in males (46, XY) a, and b - Normal chromosome from a healthy individual (controls), and the karyotype of Chromosomal aberrations in the worker group at the beginning of the experiment and after one year of exposure to X-ray,  c-Dicentric chromosome, d- chromosome break 5,6,7, and Acentric chromosome 1, e- chromatid gaps 1, 4, and 11, f- Ring chromosome 2,3,16 and chromosome break 5, and 7, g1 Dicentric chromosome, and Ring chromosome g2, h- Acentric chromosome 1, chromosome break 2, and 10, i-Chromatid gap 1, 5, and 19, chromosome break 15, and 21.

The present study shows a low percentage of chromosomal aberrations in the control group. Table 4 shows the percentage values of structural chromosomal aberrations in workers more than in controls (Figure 1a). Those abnormalities were dicentric chromosome 3%, acentric chromosome 15%, chromatid gaps 2%, and ring chromosome 10% (Table 4). The percentage of chromosome breaks in workers was very high (15%) compared to the control (0%). The result revealed no chromosome breaks and chromosome interchanges in the control group Table 4. At the same time, the abnormalities of chromosomes at the beginning of exposure to X-rays for the worker group were dicentric chromosome (6%) (Figure 1c), acentric chromosome 16% (Figure 1d), chromatid gaps 1% (Figure 1e), a chromosome breaks 15% (Figures 1d, 1f) and ring chromosome 8% (Figure 1f), but there was no chromosome interchange (Table 4). 

In addition, the comparisons of damaged cells and the percentage of chromosomal aberrations between the worker after one year of radiation exposure and a control group were shown in Table 4. The result shows highly significant differences at (P<0.0001) in damaged cells between the control and workers group with a mean of 1.79 ± 0.61 and 2.95 ± 0.91, respectively. Moreover, the percentage values of structural chromosomal aberrations in workers after exposure to X-rays for one year were more than those found in the control group (Figure 1b). The abnormalities in workers were dicentric chromosome 24% (Figure 1g), acentric chromosome 25% (Figure 1h), chromosome breaks 15% (Figures 1h, 1i), chromatid gaps 1% (Figure 1i), and ring chromosome 10% (Figure 1g), but there was no change in chromosome interchange (Table 4).

Cytogenetic analysis was performed on the peripheral blood of all patients who underwent cardiac catheterization procedures in both sexes with different doses of 124-2588 mGy at age 31-58 years. The healthy individuals (n=32) were taken as a control. The differences in damaged cells between patient groups before exposure to X-rays (before operation) and the control group were non-significant at (p=0.9958) with a mean of 1.42 ± 0.67 and 1.42 ± 0.54, respectively (Table 5).

**Table 5:** Comparisons of damaged cells and percentage of chromosomal aberrations between control and patients (before radiation) Any cell containing one or more chromosome aberrations is counted as one damaged cell.

Chromosomal aberrations	Study groups		
	Control (n=32)	Patients (n=45)	P-value
Dicentric Chromosome	4	16	
Acentric Chromosome	26	33	
Chromatid Gap	2	0	
Chromosome break	0	7	
Chromosome interchange	0	0	
Ring Chromosome	17	13	
Damage cells (%) mean (SD) Range	1.42 (0.67) 1-4	1.42 (0.54) 1-4	0.9958

**Figure 2:**
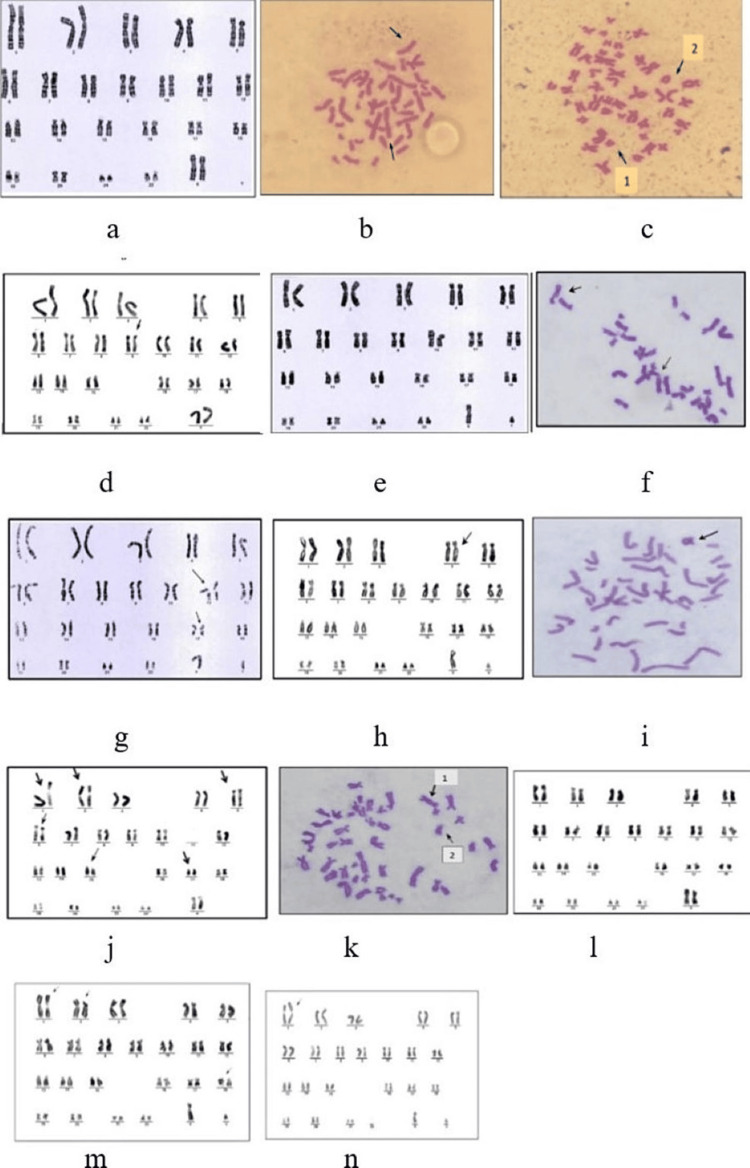
Chromosomal aberration in the control group at the beginning of the experiment Karyogram and metaphase of structural abnormality in females (46, XX) and males (46, XY) In females: a- Normal chromosome from a healthy individual (controls), b- Dicentric chromosome, c1 Acentric chromosome, and Ring chromosome c2, d- chromatid gaps 9. In males:e- Normal chromosome from a healthy individual (controls), f- Dicentric chromosome, g- Acentric chromosome 11, and 17, h- chromatid gaps 4, i- Ring chromosome.  B) in the patient group before exposure to x-ray Karyogram and metaphase of structural abnormality in females (46, XX), j- Acentric chromosome 1,2, and 6, and chromosome breaks 5, 15, k1 Dicentric chromosome, and k2 chromosome breaks, l- Ring chromosome15. In males (46, XY), m- Ring chromosome 18, Acentric chromosome 1, and chromosome break 2, n- Acentric chromosome 1.

Table 5 shows the percentage values of structural chromosomal aberrations in patients more than in controls. The percentage values of structural chromosomal aberrations in control were dicentric chromosome 4%, (Figures 2b, 2f), acentric chromosome 26% (Figures 2c, 2g), chromatid gaps 2% (Figures 2d, 2h), and ring chromosome 17% (Figures 2c, 2i) while there was no chromosome breaks and chromosomes interchange in the control group (Table 5). As well as the percentage values of structural chromosomal aberrations in the patient group before X-ray exposure were dicentric chromosome 16% (Figure 3b), acentric chromosome 33% (Figures 2j, 2m, 2n), a chromosome breaks 7% (Figures 2j, 2k, 2m), and ring chromosome 13% (Figures 2l, 2m), but there was no chromosome interchange and chromatid gaps (Table 5).

Furthermore, the differences in damaged cells between patients and worker groups before exposure to X-rays were significant at (p=0.0104) with a mean of 1.55 ± 0.60 and 2.42 ± 1.30, respectively (Table 6). The percentage values of structural chromosomal aberrations were dicentric chromosome 3%, acentric chromosome 22%, a chromosome breaks 1%, and ring chromosome 1%, while there are no chromatid gaps and chromosomes interchange for the patient group (Table 6). Whereas the abnormalities before exposure to X-rays in the worker group were dicentric chromosome 6%, acentric chromosome 16%, chromatid gaps 1%, chromosome breaks 15%, and ring chromosome 8%, but there was no chromosome interchange (Table 6).

**Table 6:** Comparisons of damaged cells and percentage of chromosomal aberrations between patients and workers before and after exposure to X-ray. Any cell containing one or more chromosome aberrations is counted as one damaged cell.

Chromosomal Aberrations	Study groups			
	Before exposure to X-ray		After exposure to X-ray	
	Patients (n=20)	worker (n=19)	Patients (n=20)	Worker (n=19)
Dicentric Chromosome	3	6	26	24
Acentric Chromosome	22	16	40	25
Chromatid Gap	0	1	0	1
Chromosome break	1	15	9	15
Chromosome interchange	0	0	2	0
Ring Chromosome	1	8	12	10
Damage cells (%) mean (SD)	1.55 (0.60)	2.42 (1.30)	4.6 (2.74)	2.95 (0.91)
P-value	0.0104		0.0170	

Table 6 shows the differences in damaged cells between patients and worker groups after exposure to X-rays. These differences were significant at (p=0.0170) with a mean of 4.6 ± 2.74 and 2.95 ± 0.91, respectively. Whereas the percentage values of structural chromosomal aberrations were dicentric chromosome 26%, acentric chromosome 40%, chromosome breaks 9%, chromosomes interchange 2%, and ring chromosome 12%, while there are no chromatid gaps in the patient group after the operation (Table 6). The result of the present study revealed that the chromosomal aberrations in workers after one year of exposure to X-ray were as follows: dicentric chromosome 24%, acentric chromosome 25%, chromatid gap 1%, chromosome break 15%, and ring chromosome 10%, but there was no chromosome interchange (Table 6).

## Discussion

Scattered radiation occurs when primary radiation interacts with the patient and is redirected in various directions and it is a significant concern, and efforts to minimize both patient and staff exposure are crucial. Personal protective equipment, such as lead aprons, plays a vital role in shielding healthcare workers from scattered radiation in preventing occupational hazards; radiation protection is the field in which the precautionary principle of As Low As Reasonably Achievable (ALARA) was first applied. It is one of the basic principles of protection against ionizing radiation [12]. The present results disagree with the studies [15,16] that did not show a significant difference between the X-ray absorbed by workers after two years of experience.

The statistical analysis for comparisons between the hematological parameters of the study groups before and after one year of the experiment showed significant differences in hematological parameters. Some of them were increased and others were decreased, but within the normal range. In contrast, slight changes in other blood parameters are not significant. This is an expected result because these individuals were not affected by any factor that led to significant changes in hematological parameters. Furthermore, the result of the present study can be explained by the fact that the workers in the catheterization lab were not previously exposed to any substance that affected the hematological parameters as compared with the control. The present results show a behavior that is consistent with the results of Ismail et al. [17] and Hamzah et al. [18]. Moreover, these results are not consistent with reported investigations [9] in Iran that showed a linear relationship between exposure doses and blood parameters of staff working in catheterization laboratories who were exposed to continuous radiation.

Although the workers in the catheterization laboratory were exposed to radiation over a period of one year, the effect on hematological parameters will not appear after comparison with the control. This may be due to two reasons: either the radiation dose of mean 5.034±0.860 mSv will not reach an amount that affects these factors, or workers take means or methods to protect themselves from the effects of radiation such as lead shielding and reducing exposure time according to ALARA which means avoiding exposure to radiation that has no direct benefit to you, even if the dose is small. To do this, they can use three basic radiation safety precautions: time, distance, and shielding [19]. Other researchers confirm that the body tissue differs in their sensitivity and extent of response to influencing factors. Blood tissue may be insensitive to this type of radiation within these conditions. The results of this study are consistent with the results of Hamza et al. [18] and Jang et al. [20] they reported that all the hematological parameters statistically non-significant differences were recorded between medical radiographers and controls.

Perhaps the reason for not being affected is that the X-ray dose absorbed by workers will not reach the level of effect on the previous hematological parameters. Various studies have shown a variety of hematologic changes. The number of RBCs was not different in X-ray-exposed individuals and controls [14,16] which agrees with the current study. The results of the present study are not consistent with Ismail et al. and Dimitrovski et al. studies, which reported WBC counts were lower in individuals exposed to X-ray this may be due to the long period of exposure. Moreover, other studies reported that WBC did not change significantly in individuals exposed to X-rays [16,20], which agrees with the current study. Furthermore, several studies [18,19] disagree with the current study that found the platelet was decreased in the radiation field workers with increased duration of exposure when compared to controls, Whereas the result of the current study agrees with [16,18]. Those studies reported that the platelet and white blood cell counts have not changed. Mean corpuscular hemoglobin (MCH), hematocrit, and red cell distribution width (RDW) values have been reported to be lower [19] or higher [13,18] in X-ray exposure which is not consistent with the current study. Lymphocyte and granulocyte counts tend to be lower in X-ray workers [14,15], which disagrees with the current study. The lymphocyte count was higher in studies of ISCN, and Mousavikia et al. [13,16] which disagrees with the present study. Because chronic exposure to X-rays can produce alteration in hematological parameters, some parameters significantly decrease and some of them significantly increase [20]. On the other hand, both patients and comparison subjects have the same hematological parameters the reason may be the patients had not previously been exposed to any external or internal factors that led them to find a difference between them and the control. The results of this study are largely consistent with the [12,14]. On the other hand, both the short time and low doses may be the reason why all patients remain unaffected after the operation. The findings of this study are consistent with the study of ICRP [12].

The comparisons between hematological parameters for patients and workers before exposure to radiation in male cases showed significant differences in some hematological parameters and non-significant differences in others but within the normal range. Firstly, the reason for this can be attributed to the fact that cath lab workers have never been exposed to high doses of X-ray and other influencing factors on their blood tissue throughout their work in the cath lab and their life in general, and in contrast, patients have also not been exposed to such influential factors. Finally, this result confirms that the exposure period and the amount of radiation to which both samples were exposed in the current study will not reach a state of influence on blood tissue. As well as, the radiation effects of the hematopoietic tissue are related to the total absorbed dose and the dose or frequency of repeated exposure [12,13]. The data concerning the effects of variables on dosage and the accumulated dosage is sparse. In general, a significant dose-dependent decrease in red blood cell counts has been reported after ionizing radiation [19]. Some studies indicated that red blood cells tend to recover after several days or weeks of radiotherapy for example, and signs of improved hematopoietic repair after chronic radiation are recorded [13]. A study by Faraj and Mohammed in Japan asserted that upon radiation exposure, blood cell counts begin to drop and then rebound [21].

In addition, the comparisons of damaged cells and the percentage of chromosomal aberrations between the study group were highly significant differences between the control and workers group after exposure to X-rays for one year. The abnormalities in workers were dicentric, acentric chromosome, chromosome breaks, chromatid gaps, and ring chromosome, but there was no change in chromosome interchange. According to previous studies, the results agree with research carried out in other countries such as Iran and Kazakhstan [20,22]. Dicentric chromosomes have been identified as instigators of the genome instability associated with cancer, but this instability is often resolved by one of several different secondary events. These include centromere inactivation, inversion, and intercentromeric deletion [21].

The present study demonstrates that the inductions of dicentrics, acentric, chromosome break, and ring chromosomes in human lymphocytes were intimately related to the irradiation dose. Therefore, these cytogenetic analyses will be helpful for an estimation of radiation exposure dose. These outcomes agree with investigations reported by Ryu et al., [23] and Sanzari et al. [24].

Ionizing radiation can induce specific types of chromosome aberrations. Acute radiation response occurs mainly in renewal tissue and has been related to the death of critical cell populations such as the stem cells in the bone marrow. These responses occur within three months of the start of radiation treatment or exposure but are not usually limiting for fractional radiation treatment because of the ability of the tissue to undergo rapid repopulation to regenerate the parenchymal cell population [24]. The use of chromosome aberrations as biomarkers for accurate radiation dose reconstruction at the individual level is uncertain; however, high levels of chromosome aberrations apparently indicate potential risk. Therefore, chromosome aberrations can likely be used as an effective early marker of radiation exposure at the population level in long-term follow-up studies.

The results of the present study revealed a high percentage of dicentric chromosomes in workers compared with controls. The present results show a behavior that agrees with the findings of Jang et al. [20] and Sanzari et al. [24] and come back identical to Suzuki et al. [22] and Sanzari et al. [24].

Limitations

The present study has certain limitations. The data obtained from subjects working in the cardiac catheterization unit may not be represented in other X-ray units, due to the difference in X-ray exposure time and because our main goal is to determine the effects of X-ray on the hematological parameters and genetic material (damaged cells) in individuals those are working in the catheterization unit and patients underwent cardiac catheterization process, therefore all data collected only from each individual worker in Azadi Teaching Hospital Heart Center in Duhok, Kurdistan region, Iraq and patients those underwent the same cardiac catheterization unit.

## Conclusions

The results of the present study showed that the scattered X-rays in the catheterization unit after the end of the experiment did not change significantly. The current study also revealed that all hematological parameters in the blood of workers and patients in the catheterization unit did not change significantly after exposure to X-rays, whereas the damaged cells (chromosomal aberrations) in patients did not change significantly compared with the control group at the beginning of the experiment. In patients, these cells were increased after the operation, but were present at a high level in the workers, as compared with controls. The damaged cells in workers remained constant from the beginning of the experiment till the end. Finally, patients had increased damaged cells after the end of the trial period compared to workers. However, given the results of this study and antithetical results reported in various studies, it is recommended to undertake studies with larger sample sizes and longer duration.

According to the present results, it is obvious to assume that the occupational dose of interventional cardiologists and cardiac catheterization workers tends to be higher compared to other medical specialists as a result of the recent increasing use of interventional techniques. On the other hand, physicians are dramatically unaware of the dose, long-term risks, and population health impact caused by the use of medical ionizing radiation. Thus, a major awareness appears to be crucial to improve one's knowledge of the appropriateness of protective tools and also in trying to reduce the number of unnecessary procedures. The use of a biological dosimeter could be a reliable tool for risk quantification on an individual basis.
